# The mediating role of loneliness in the relationship between smartphone addiction and subjective well-being

**DOI:** 10.1038/s41598-024-54546-3

**Published:** 2024-02-23

**Authors:** Peng Su, Mu He

**Affiliations:** 1 School of Marxism, University of Chinese Academy of Social Sciences, Beijing, China; 2 College of Marxism, Chongqing Medical and Pharmaceutical College, Chongqing, China

**Keywords:** Subjective well-being, Martphone addiction, Loneliness, Mediating role, College students, Spiritually prosperity, Psychology, Human behaviour

## Abstract

As smartphones become increasingly prevalent worldwide, the relationship between smartphone addiction and subjective well-being has become a focal point in academic circles. Prior research predominantly delved into the direct correlation between smartphone addiction and subjective well-being, yet there remains a dearth in exploring its underlying mechanisms. This study investigated the mediating role of loneliness in the relationship between smartphone addiction and subjective well-being among Chinese university students. Conducted across 16 universities in eight provinces and municipalities in China, this study encompassed 1527 university students. Data collection utilized scales measuring smartphone addiction, loneliness, and subjective well-being. The findings revealed that: (1) demographic variables such as place of origin, educational level, and family income influenced university students' subjective well-being; (2) a significant negative correlation existed between smartphone addiction and subjective well-being among university students, coupled with a significant positive correlation between smartphone addiction and loneliness, indicating the significant negative predictive effect of smartphone addiction on subjective well-being; (3) loneliness partially mediated the relationship between smartphone addiction and subjective well-being among university students, suggesting that smartphone addiction could directly impact university students' subjective well-being, or indirectly through its effect on loneliness.

## Introduction

Subjective well-being refers to an individual's assessment of their life circumstances and quality, encompassing cognitive judgments such as life satisfaction and the presence of positive or negative emotions^1^. It significantly influences the mental and physical health of university students. Previous research has revealed a negative correlation between subjective well-being and anxiety/depression, indicating lower levels of anxiety and depression with higher subjective well-being^2^. Additionally, the pursuit of happiness and a meaningful life serves as a protective factor against suicide, reducing the risk among university students^3^. Moreover, studies have found that youth with higher levels of subjective well-being tend to achieve better academic performance and academic success^4^. This underscores the importance of subjective well-being in the healthy development of university students. Numerous factors influence the subjective well-being of university students. Existing research has identified social support^5^, social relationships^6^, sudden major public health events and associated lockdown policies^7^, optimistic personality traits^8^, sleep quality, resilience^9^, socioeconomic status, physical activity, and even geographical and cultural environments^10,11^, all significantly correlating with an individual's subjective well-being. With the evolution of information technology, the impact of technological devices such as smartphones on subjective well-being has emerged as a recent hot topic. However, academia has shown limited research on the relationship between smartphone addiction and the subjective well-being of university students, lacking a deeper exploration of their intrinsic connection.

### Smartphone addiction and subjective well-being

Smartphone addiction refers to compulsive behavior characterized by physiological, psychological, emotional, and social dysfunction due to excessive reliance on smartphones^12,13^, primarily manifested as lack of self-control and excessive smartphone use^14^. Current research focuses on the relationship between specific addictive behaviors during smartphone usage and subjective well-being, such as addiction to different software or various forms of entertainment. In terms of software addiction, studies have found a negative correlation between Facebook (now renamed Meta) addiction and college students' life satisfaction and positive emotions^15^. Additionally, temporary abstinence from the social media app Instagram led to increased subjective well-being among young women^16^. Regarding entertainment addiction, research has shown a negative correlation between online gaming addiction and university students' subjective well-being^17^. Similarly, addiction to short videos was found to negatively correlate with college students' happiness^18^. Analysis indicates a significant correlation between addictive behaviors resulting from smartphone usage and subjective well-being. However, there is limited specific research on the relationship between smartphone addiction and the subjective well-being of university students. Some studies suggest that adolescents with longer and more frequent smartphone usage experience higher subjective well-being^19^. However, conflicting results have also emerged, with analyses indicating a negative correlation between smartphone addiction and the subjective well-being of university students^20^. As a result, the relationship between smartphone addiction and the subjective well-being of university students remains contentious, warranting further exploration for its substantial real-world implications. Hence, the hypothesis is proposed:

#### Hypothesis 1

Smartphone addiction is expected to be negatively correlated with the subjective well-being of university students.

### Mediating role of loneliness

Loneliness is a naturally occurring subjective assessment^21,22^, depicting an individual's psychological state arising from the disparity between expected and actual social interaction within the societal framework^23^. In studies regarding loneliness and smartphone addiction, the majority of research findings indicate a positive correlation between smartphone addiction and loneliness^24–26^. As smartphone addiction intensifies, individuals experience heightened feelings of loneliness^27^. However, there are studies suggesting a negative correlation between smartphone addiction and individual loneliness^28^, a U-shaped relationship^29^, or even non-significant correlations^30^. Regarding research on loneliness and subjective well-being, studies have found a negative correlation between loneliness and college students' life satisfaction^15^. Additionally, loneliness has been identified to longitudinally predict individual subjective well-being^31^. Simultaneously, various studies have found significant correlations between loneliness and subjective well-being among different demographics such as elderly individuals^32^, adolescents^33^, and college students^15,34^. Analysis demonstrates a significant correlation between loneliness and both smartphone addiction and subjective well-being. However, there is limited analysis in current research exploring the mediating role of loneliness between smartphone addiction and subjective well-being among university students. Hence, investigating the mediating role of loneliness between smartphone addiction and subjective well-being among university students holds significant innovation potential and can address the gaps in existing research. Based on this, the hypothesis is proposed:

#### Hypothesis 2

Loneliness is expected to mediate the relationship between smartphone addiction and subjective well-being among university students.

## Methods

### Participants

Through stratified cluster sampling, 1527 valid questionnaires were collected from 16 universities across 8 provinces and municipalities nationwide. The substantial sample size and its broad representation render the sample adequately representative. Among the 1527 participants, there were 1000 females (65.49%) and 527 males (34.51%). The educational distribution comprised 390 participants with an associate degree (25.54%), 971 with a bachelor's degree (63.59%), and 166 pursuing postgraduate studies (10.87%), with ages ranging from 17 to 40 years. From the participants, 855 hailed from rural backgrounds (55.99%), while 672 came from urban settings (44.01%).

### Measurement tools

#### Smartphone addiction scale

The Smartphone Addiction Tendency Scale for College Students developed by Xiong et al.^35^was used in this study. The scale consists of 16 items grouped into four factors: withdrawal symptoms, salience, social comfort, and mood changes. Each item is rated on a 5-point scale, with higher scores indicating a greater tendency towards Smartphone addiction. The Cronbach's α coefficient for this scale in this study was 0.84.

#### Loneliness scale

The third version of the Loneliness Scale developed by Russell et al.^36^was used in this study. The scale consists of 20 items and measures a single factor: loneliness. Each item is rated on a 4-point scale, with higher scores indicating a stronger feeling of loneliness. The Cronbach's α coefficient for this scale in this study was 0.91.

#### Subjective well-being scale

The Subjective Well-Being Scale developed by Campbell^37^ was used in this study. The scale consists of nine items grouped into two factors: overall emotional index and life satisfaction. Each item is rated on a 7-point scale, and the weighted sum of the two factors yields the overall subjective well-being score. Higher scores indicate a higher level of subjective well-being. The Cronbach's α coefficient for this scale in this study was 0.89.

### Procedures

This study employed a stratified cluster sampling method. From January to May 2022, 16 universities across 8 provinces and municipalities in China were selected as primary sampling units. Within each university, 2–3 colleges were further selected as secondary sampling units for cluster sampling. With the cooperation of relevant institutions and faculty, a total of 1527 valid questionnaires were collected. The survey was conducted anonymously, and participants were required to provide informed consent before completing the questionnaire. Following questionnaire collection, this study conducted fundamental analyses using SPSS 26.0, including independent samples t-tests, one-way analysis of variance (ANOVA), correlation analyses, and mediated effect examinations utilizing the PROCESS v4.0 plugin developed by Hayes^38^. Specifically, the Bootstrap method was employed, involving 5000 iterations to create a 95% confidence interval for the samples. Ethical approval for this study was obtained from the Ethics Committee of Chongqing Medical and Pharmaceutical College, adhering to the requirements outlined in the Helsinki Declaration.

## Results

### Common method bias test

To reduce the possible influence of common method bias, anonymous questionnaire and different scoring methods were adopted. The Harman single-factor test was used to examine common method bias, with an unrotated factor analysis showing that nine factors had eigenvalues greater than 1, with the first factor explaining 20.61% of the variance, which is less than the critical value of 40%^39^. Therefore, this study did not have significant common method bias.

### Differences in subjective well-being and demographic variables

The investigation revealed that the subjective well-being score among university students was 5.50 ± 1.57. Independent sample t-tests and one-way ANOVA were conducted on relevant variables. Concerning the variable of students' origins, urban students exhibited significantly higher subjective well-being (5.66 ± 1.62) compared to rural students (5.38 ± 1.51), with a statistically significant difference (*P*< 0.001). Regarding educational levels, undergraduates' subjective well-being (5.58 ± 1.57) was slightly higher than that of college students (5.32 ± 1.56) and postgraduates (5.47 ± 1.53), with statistical significance (*P*< 0.05). Regarding family income, students with a per capita monthly income above 4000 yuan displayed significantly higher subjective well-being (5.85 ± 1.68) compared to those with 4000 yuan or below (5.17 ± 1.37), showing statistical significance (*P*< 0.001). However, differences in subjective well-being among students of different genders did not reach statistical significance (*P*> 0.05).

### Analysis of the relationship between smartphone addiction, loneliness, and subjective well-being

Through correlation analysis, the relationship between smartphone addiction, loneliness, and subjective well-being was explored (see Table1). It was found that there was a significant negative correlation between subjective well-being and smartphone addiction as well as loneliness. There was also a significant positive correlation between smartphone addiction and loneliness, which supports Hypothesis 1.

**Table 1:** Correlation analysis of smartphone addiction, loneliness and subjective well-being (*r*).

Variables	***x***± s	Smartphone addiction	Loneliness	Subjective well-being
Smartphone addiction	38.02 ± 7.83	1		
Loneliness	40.23 ± 10.53	0.205***	1	
Subjective well-being	5.50 ± 1.57	-0.284***	− 0.306***	1

***p < 0.001, the same below.

### The mediating role of loneliness

**Figure 1:**
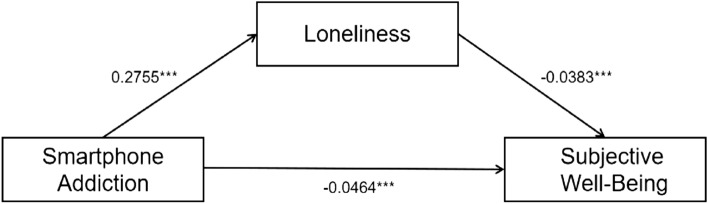
Mediating effect model.

Based on pertinent theoretical research, demographic variables exhibiting statistical differences were included as control variables in a regression model to establish a mediation model of smartphone addiction affecting subjective well-being through loneliness. Additionally, the PROCESS plugin was employed to examine the mediating effects. The analysis results (refer to Tables2,3, Fig.1) demonstrate that smartphone addiction negatively predicts subjective well-being (β = − 0.0569,*P*< 0.001). Even after incorporating the mediating variable into the regression model, smartphone addiction maintains a significant negative predictive effect on subjective well-being (β = − 0.0464,*P*< 0.001). Smartphone addiction significantly positively predicts feelings of loneliness (β = 0.2755,*P*< 0.001), while loneliness significantly negatively predicts subjective well-being (β = − 0.0383,*P*< 0.001). The direct effect of smartphone addiction on subjective well-being, along with the mediation effect through loneliness, both show 95% confidence intervals in the bootstrap test that do not include zero. The mediation effect accounts for 18.45% of the total effect, indicating that smartphone addiction can directly and indirectly predict subjective well-being through the mediation of loneliness. Hypothesis 2 is supported by the analysis.

**Table 2:** Test of mediation model of loneliness.

	Subjective well-being		Loneliness		Subjective well-being	
	β	*t*	β	*t*	β	*t*
Place of origin	0.1112	1.3776	− 0.6658	-1.1812	0.0857	1.1012
Educational level	0.0442	0.6684	− 0.7700	-1.6676	0.0147	0.2309
Per capita monthly household income	0.2232	7.0734***	0.1078	0.4893	0.2273	7.4729***
Smartphone addiction	− 0.0569	− 11.7987***	0.2755	8.1797***	-0.0464	-9.7603***
Loneliness					-0.0383	-10.8137***
R^2^	0.1182		0.0451		0.1811	
F	50.9931		17.9748		67.2893

**Table 3:** Analysis of total effect, direct effect and mediating effect.

	Effect	BootSE	BootLLCI	BootULCI	Effect ratio (%)
Total effect	− 0.0569	0.0049	− 0.0665	− 0.0472	
Direct effect	− 0.0464	0.0049	− 0.0557	− 0.0367	81.55
Mediating effect of loneliness	− 0.0105	0.0015	− 0.0137	− 0.0077	18.45

## Discussion

This study focuses on the subjective well-being of university students, analyzing the correlation between smartphone addiction and their subjective well-being, and further exploring the underlying mechanisms of these two variables. Previous research has often been limited to specific subsets of university students—be it undergraduates, graduates, or college students—and primarily concentrated on specific addictive behaviors related to smartphone use (such as gaming addiction). There has also been a lack of exploration into the mediating factors between smartphone addiction and subjective well-being. This study addresses these limitations observed in prior research. We have uncovered some findings that warrant further discussion: (1) The subjective well-being of university students is influenced by demographic variables like their place of origin, educational level, and family income. (2) Smartphone addiction is negatively correlated with subjective well-being and positively correlated with feelings of loneliness. (3) Loneliness acts as a mediator between smartphone addiction and the subjective well-being of university students. This study fills in the gaps of previous research to some extent and provides a foundation for a deeper understanding of the relationship between smartphone addiction and the subjective well-being of university students.

The first finding suggests that subjective well-being is higher among urban university students compared to their rural counterparts. Additionally, undergraduate students exhibit significantly higher subjective well-being than both college and graduate students. Moreover, subjective well-being is higher among students from families with higher average incomes than those from lower-income families. There were no significant differences in subjective well-being between male and female university students. Regarding the place of origin, the observed difference in subjective well-being between urban and rural university students aligns with prior research^40^. This difference might be attributed to the reduced familial support available to rural students, contributing to lower resilience during major health crises and a decrease in quality of life, resulting in diminished subjective well-being. In terms of educational levels, the higher subjective well-being among undergraduate students in contrast to college and graduate students diverges from previous studies^17^. This discrepancy could stem from this study's national scope compared to previous region-specific research. Furthermore, the inclusion of not only undergraduates and college students but also graduate students might have influenced the outcomes. Regarding family income, the stronger subjective well-being among university students from higher-income families aligns with past studies^41,42^. This could be due to lower survival pressures, greater material abundance, reduced negative emotions, and higher life satisfaction among students from higher-income households. While some studies propose that income doesn't always directly impact happiness and might change over time^43^, we believe this notion doesn't conflict with the conclusions of this study, as this temporal shift is a relatively gradual process. In terms of gender, the absence of significant differences in subjective well-being between male and female university students could be attributed to China's socially equal gender environment, ensuring equitable experiences in campus and family life, potentially contributing to the absence of substantial differences in life satisfaction and emotions. Prior studies analyzing smartphone addiction rarely explored the additional impact of demographic variables. The inclusion of demographic variables in this study provides a more comprehensive understanding of the factors influencing the subjective well-being of university students.

The second finding indicates that the higher the degree of smartphone addiction, the lower the subjective well-being, supporting Hypothesis 1. This aligns with the majority of previous research findings^15,16,18,20^. The Habit Formation Theory provides a sound theoretical explanation, suggesting that individual habits result from repetitive behaviors and maintaining these habits influences people's quality of life and satisfaction^44^. Smartphone addiction, being a habitual behavior developed through prolonged repetition, often correlates with unhealthy habits such as staying up late, lack of outdoor activities, fostering anxiety, depression, and other psychological issues^12,13^. This adversely affects both physical and mental health, resulting in reduced quality of life and lower subjective well-being^45^. Moreover, the advancement of new technologies often sparks widespread attention towards the pursuit of a happier life^46^, further fueling smartphone addiction. Although technological habits formed by smartphones might temporarily enhance an individual's sense of well-being^19^, once reaching an excessive addictive level, they may be associated with a decline in subjective well-being. This study further confirms the negative correlation between smartphone addiction and the subjective well-being of university students, addressing past controversies surrounding the relationship between smartphone addiction and subjective well-being. Simultaneously, the second finding illustrates that a higher level of smartphone addiction corresponds to a stronger sense of loneliness, consistent with numerous previous studies^24–27^. The rationale behind this correlation might be that some of the realistic needs of university students remain unmet, prompting them to seek instant satisfaction and joy through their phones. However, smartphone addiction often brings about a sense of isolation from reality and provides a virtual, yet superficially satisfying, experience. Upon returning to reality, individuals often feel emptier and more helpless, intensifying feelings of loneliness, consequently fostering a deeper reliance on smartphones^24^. This study validates the positive correlation between smartphone addiction and feelings of loneliness, corroborating past debates regarding the relationship between smartphone addiction and loneliness.

The third finding indicates that loneliness acts as a mediating factor between smartphone addiction and subjective well-being, supporting Hypothesis 2. This aligns with previous research outcomes^15,17^. The theory of weakening offline relationships posits that excessive use of smartphones and similar online communication devices strengthens online relationships while potentially correlating with a reduction in offline social interactions. This may result in isolating individuals from their immediate environment and creating a disconnect between the real and virtual contexts^47^. This subsequently affects the quantity and quality of offline social interactions, heightening feelings of loneliness, reducing effective social support, and consequently diminishing subjective well-being^48^. With the advancement of information technology, the widespread use of communication devices like smartphones among university students can enrich their daily academic and social life, providing convenience for interpersonal communication and interaction^49^, and even potentially enhancing their sense of happiness^19^. However, smartphone addiction weakens the real-life social abilities of university students, immersing them in a virtual space detached from reality, reducing the number of friends in real life, decreasing social interaction quality, and intensifying feelings of loneliness^25,27^. Happiness tends to fluctuate over time, with actively shaping novel and surprising experiences contributing to an increase in happiness^50^. Conversely, negative experiences of increased loneliness are associated with individuals being less inclined toward engaging in real-life interactions, lacking the courage and confidence to tackle complex problems, and perceiving a lack of adequate social support^31^. These factors are potentially linked to a decline in happiness^51,52^. Prior research often focused solely on specific addictive behaviors within smartphone use (such as gaming addiction) and their relationship with subjective well-being, while lacking exploration into the intermediary factors between the two. This study innovatively explores the mediating role of loneliness between smartphone addiction and the subjective well-being of university students within the smartphone addiction domain.

## Conclusions

The present study unveils significant associations among smartphone addiction, feelings of loneliness, and subjective well-being in university students, confirming the mediating role of loneliness between smartphone addiction and subjective well-being. While smartphone use might not immediately reduce an individual's subjective well-being^15^, once addiction sets in, it may be associated with a decline in subjective well-being, exerting an indirect influence by affecting feelings of loneliness. These findings bear crucial implications for enhancing the subjective well-being of university students. Educational professionals in higher institutions can elevate the subjective well-being of students by preventing smartphone addiction. Furthermore, strategies such as bolstering familial, peer, and institutional support can mitigate loneliness among university students, thereby enhancing their subjective well-being.

### Strengths and limitations

One of the strengths of this study lies in its substantial sample size (1527), encompassing college students, undergraduates, and graduate students. However, utilizing cross-sectional data presents a limitation, suggesting future research to employ longitudinal investigation merging both longitudinal and cross-sectional data. Another limitation of this study arises from using self-reported survey data. Future studies could consider employing objective measurement tools to further enhance the scientific rigor of the research.

## Data Availability

The datasets used during the current study available from the corresponding author on reasonable request.
